# Remodeling and Control of Homologous Recombination by DNA Helicases and Translocases that Target Recombinases and Synapsis

**DOI:** 10.3390/genes7080052

**Published:** 2016-08-19

**Authors:** Sarah J. Northall, Ivana Ivančić-Baće, Panos Soultanas, Edward L. Bolt

**Affiliations:** 1 School of Chemistry, University of Nottingham, Nottingham NG7 2RD, UK; panos.soultanas@nottingham.ac.uk; 2 Department of Molecular Biology, University of Zagreb, Zagreb 10000, Croatia; ivanai@irb.hr; 3 School of Life Sciences, University of Nottingham, Nottingham NG7 2RD, UK; ed.bolt@nottingham.ac.uk

**Keywords:** homologous recombination, synapsis, helicase, Hel308

## Abstract

Recombinase enzymes catalyse invasion of single-stranded DNA (ssDNA) into homologous duplex DNA forming “Displacement loops” (D-loops), a process called synapsis. This triggers homologous recombination (HR), which can follow several possible paths to underpin DNA repair and restart of blocked and collapsed DNA replication forks. Therefore, synapsis can be a checkpoint for controlling whether or not, how far, and by which pathway, HR proceeds to overcome an obstacle or break in a replication fork. Synapsis can be antagonized by limiting access of a recombinase to ssDNA and by dissociation of D-loops or heteroduplex formed by synapsis. Antagonists include DNA helicases and translocases that are identifiable in eukaryotes, bacteria and archaea, and which target synaptic and pre-synaptic DNA structures thereby controlling HR at early stages. Here we survey these events with emphasis on enabling DNA replication to be resumed from sites of blockage or collapse. We also note how knowledge of anti-recombination activities could be useful to improve efficiency of CRISPR-based genome editing.

## 1. Homologous Recombination Linked to DNA Replication

Homologous recombination (HR) is genetic exchange between identical or similar DNA molecules that provides the basis for meiosis and various mechanisms of DNA repair. When DNA replication forks encounter DNA damage, in the form of template breaks or lesions, HR provides repair by utilizing an undamaged DNA strand to reactivate the stricken replication fork [1,2,3]. This tends to make HR mediated repair of DNA replication high fidelity at the site of damage compared to end-joining processes that result in loss of base-pairs. As discussed in this review, HR repair of DNA replication is kept under control to balance the need to re-start replication against the potential for genetic diversity that may be in this context an undesirable by-product of HR.

**Figure 1:**
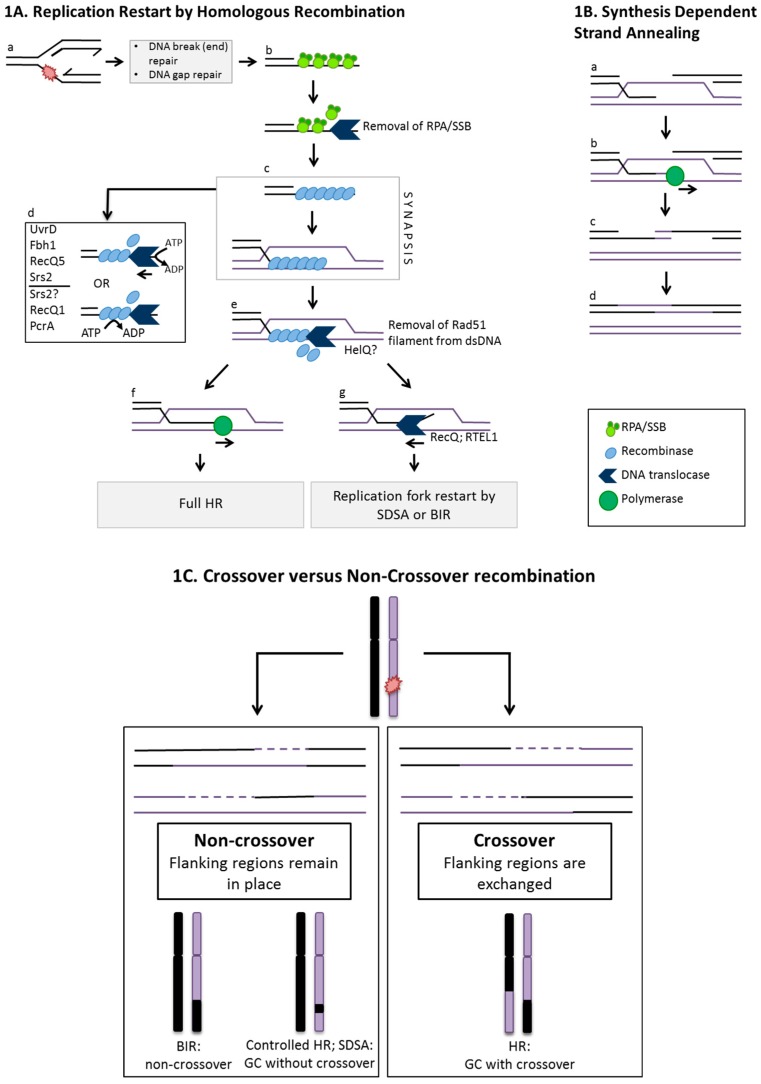
(**A**) Replication restart by homologous recombination and actions that control it at pre-synapsis or synapsis. (**a**) Several possible branched DNA structures can arise when replication forks are compromised as a result of chemical damage or protein bound DNA complexes. These include ssDNA gaps and DNA strand breaks, indicated by the red paint-splash. Exposed single stranded DNA (ssDNA) within partial duplexes is coated by binding proteins (e.g., SSB and RPA); (**b**) RPA/SSB can be removed from ssDNA by DNA helicases following replication fork stalling, to facilitate loading of recombinase monomers onto ssDNA as a nucleoprotein filament (NPF); (**c**) A recombinase NPF catalyses strand invasion into homologous duplex, aided by mediator proteins that include Rad51 paralogues, displacing the complementary strand creating a D-loop (synapsis); (**d**) The NPF may be disrupted by DNA translocases via physical displacement of the NPF by ATP dependent protein translocation or, by physical interaction between DNA translocases and the NPF monomers that activates intrinsic ATPase activity within the NPF causing filament disassembly. Various helicases have roles here, as detailed in the text and listed in the figure; (**e**) Following synapsis, the recombinase can be removed from heteroduplex DNA, for example by Hel308/ HelQ proteins; (**f**) Extension of 3′ OH invading strand by replicative DNA polymerases illustrates the interplay between nascent DNA replication and the onset of homologous recombination (HR). This can lead to Holliday junction formation and full homologous recombination toward crossover or non-crossover products (see Figure 1C); (**g**) Unwinding of the invading strand in D-loops occurs by DNA helicases such as RecQ or RTEL1 to allow for SDSA or break induced replication (BIR) that form non-crossover products; (**B**). Synthesis dependent strand annealing after synapsis. (**a**) Synapsis leads to D-loop formation in which an available 3′ OH end of the invading strand can be extended by polymerases (**b**); (**c**) The extended DNA strand arising from synapsis and replication can be displaced from the D-loop thereby preventing end capture and the later stages of HR. Nascent DNA is re-annealed, extended further (if necessary) and ligated to its original strand; (**C**) Crossover versus Non-crossover events in homologous recombination. Pathways of HR repair DNA damage in ways that can lead to different genetic outcomes in terms of genome stability. Non-crossover is the preferred outcome to maintain genetic status quo and occurs preferentially through BIR, synthesis dependent strand annealing (SDSA) and controlled HR pathways where sites flanking repaired regions remain in place. Crossover can occur following resolution of Holliday junctions where reciprocal exchange of sites flanking repaired regions takes place and can lead to chromosomal rearrangements. Gene conversion following copying of genetic information from intact homologous DNA can occur in addition during crossover and non-crossover events.

HR initiates from damaged single stranded DNA being invaded into a homologous duplex by a “strand exchange” reaction (“synapsis”) that creates a D-loop (“displacement loop”) intermediate. The D-loop structure is central to restarting DNA replication that preserves DNA sequence information from the homologous template (Figure 1A). HR may be called upon for replication repair in response to chemical damage to DNA if it inhibits replicative polymerase or helicase activities [4], or if nucleoprotein complexes block the combined actions of replicative and accessory helicases at the replisome [5]. Reactivation of blocked or collapsed replication sites remote from replication origins is possible if the fork is stabilized or processed by repair nucleases and helicases into suitable substrates for re-priming replication. Excision repair processes can deal with the DNA strand lesion. Exposure of ssDNA at compromised or collapsed replication forks makes the presence of single-stranded DNA binding proteins, such as single stranded DNA binding protein (SSB) and replication protein A (RPA), important for how repair by HR can proceed at these sites. Here we focus on how and why HR initiated from ssDNA is regulated as part of DNA repair processes.

## 2. Controlling Homologous Recombination Choices at D-loops

Because HR is initiated from a synaptic D-loop this structure can be viewed as a crucial checkpoint for whether or not HR is allowed to proceed. This review is primarily focused on processing of the D-loop during these checkpoint choices, and the events immediately prior to D-loop formation (pre-synapsis), in which invading ssDNA is loaded with a recombinase enzyme to form a nucleoprotein filament (NPF). A wider perspective on cellular events regulating HR in bacteria and eukaryotes can be found elsewhere, for example in [6,7]. We here briefly review factors that help to promote HR by aiding recombinases to form NPFs and D-loops, and then consider in more detail; (a) processes that banish HR by promoting replication fork re-activation prior to synapsis, (b) processes that disassemble pre-synaptic NPFs, therefore inhibiting HR, and (c) enzymes that regulate synapsis by dissociating D-loop or heteroduplex products. The roles of helicases/translocases feature prominently in each of these processes, achieving their regulatory effects on HR through a variety of ATP-dependent and ATP-independent mechanisms.

D-loops formed in synapsis facilitate nascent DNA synthesis, through the heteroduplex DNA region, by strand extension from the 3′ OH end of the invading DNA (Figure 1A). Thereafter several possible outcomes generate products in which the newly replicated DNA is a common factor (Figure 1A,B). The different outcomes of HR highlight how it can generate genetic diversity through chromosomal re-arrangements, as well as maintain genome integrity by accurate DNA repair. Genetic diversity arises from HR proceeding into late stages, forming DNA crossover products (Figure 1C). This may be desirable in certain circumstances (e.g., meiosis), but is not desirable for completion of accurate DNA repair because it is associated with provoking cancers and inherited genetic diseases; readers are referred elsewhere [8,9] for full explanations of how HR proceeds into late stages that include branch migration and Holliday junction resolution that characterize routes to crossover or non-crossover products. Alternatively, crossing over can be avoided by directing DNA repair into routes generating only non-crossover products. Opportunities for this arise at early stages of HR, especially during synapsis in which D-loops may be processed into pathways referred to as synthesis dependent strand annealing (SDSA) and break induced replication (BIR) [10] (Figure 1B). SDSA and BIR can both also utilize synapsis to facilitate nascent DNA synthesis prior to diverting HR away from branch migration and Holliday junction formation [11]. Therefore, it is clear that pre-synapsis, synapsis and D-loops all represent critical checkpoints for determining if, and how far, HR will proceed.

**Figure 2:**
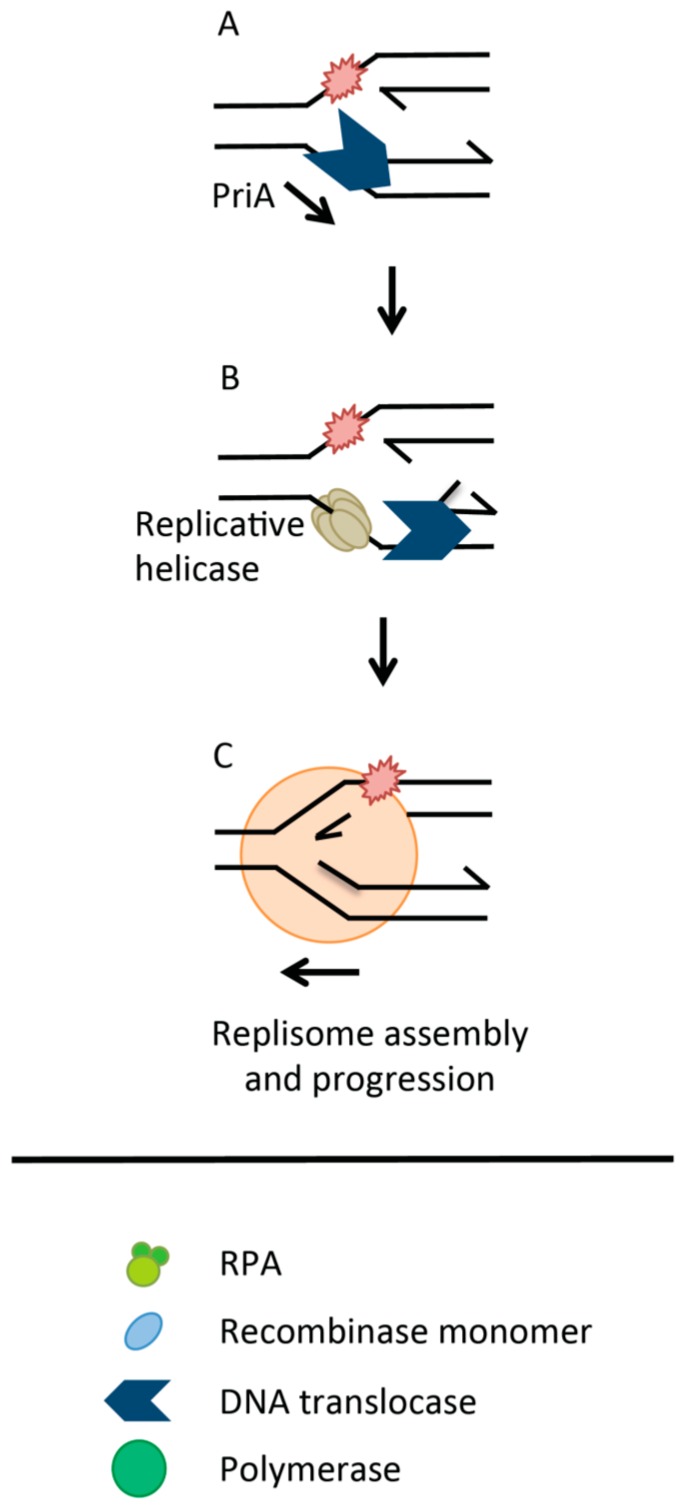
Direct Replication Restart. (**A**) PriA * recognizes branched DNA structures arising at stalled replication forks. It remodels forks to present substrates for binding by replicative helicase loading proteins PriB and PriC; (**B**) Recruitment of the replicative helicase DnaB for loading onto the lagging strand template allows replication to be re-activated; (**C**). * Protein nomenclature here refers to *E. coli*.

The need for regulation of HR as a repair process could be removed completely if recombination were avoided totally, by re-activating replication using alternative means (Figure 2). Depending on the nature of the replication impediment, non-recombinogenic processes could be utilized in the form of translesion polymerases [12,13] and possibly traversing helicases like FancM [14]. Blocked replication forks may also be re-activated without recombination, for example in bacteria through fork remodeling activities of proteins such as PriA, which reload the replisome at sites other than origins and independently of replicative helicase loaders [15,16,17,18] (Figure 2). Activity of direct replication restart is itself counter-balanced by regulatory helicases such as RecG [19,20], reviewed extensively in [21,22]. Interplay between excision repair systems and “Rec” proteins may also restore replication by mechanisms that involve RecFOR and the bacterial recombinase, RecA [23].

## 3. Recombination Initiated from ssDNA: Nucleoprotein Filaments and D-loops

Strand exchange reactions (synapsis) are universally catalysed by recombinase enzymes of the RecA family that are commonly called RecA in bacteria, Rad51 in eukaryotes, and RadA in archaea [24]. Most sequence similarity between recombinases resides within essential Walker A and B motifs that confer ATPase activity. Recombinase catalysed synapsis reactions are relatively well studied by biophysical, structural, biochemical and genetic analyses. To be able to understand molecular mechanisms of how synapsis is regulated it is important to unravel details of recombinase structure-function when interacting with DNA and other proteins that promote NPF formation; a brief overview of the major players is given here, and interested readers are directed to comprehensive reviews of the topic [25,26].

In pre-synapsis, recombinase monomers (RecA, Rad51 or RadA) assemble cooperatively onto ssDNA forming a right-handed NPF that extends DNA length by about 150% comparatively to B-form DNA, in the case of RecA, facilitating the homology search immediately prior to synapsis. Recombinase filaments are dynamic, using cycles of ATP binding and hydrolysis to mediate high affinity DNA binding (ATP-bound) and dissociation (ADP-bound). NPF formation therefore requires that recombinases gain access to ssDNA that is pre-bound with single-stranded DNA binding proteins such as RPA or SSB (Figure 1A). In bacteria, RecBCD and RecF recombination systems assist with loading RecA onto ssDNA to initiate HR by D-loop formation. RecBCD and RecF recombination systems use different sets of enzymes and substrates to produce 3′ ended ssDNA for loading with RecA. RecBCD is multifunctional enzyme that binds to dsDNA ends, activating highly processive helicase activity and ATP-dependent ds- and ss-DNA exonuclease activities [27,28]. RecBCD’s helicase, nuclease and RecA loading activities (and therefore initiation of HR) are regulated by hotspots of recombination called Chi sites (5′-GCTGGTGG-3′) which promote recombination in their vicinity [29,30], reviewed in [31]. Prior to interaction with Chi, RecBCD binds to dsDNA ends and unwinds and degrades mostly the 3′ ended strand. After Chi recognition, digestion is targeted to the 5′ ending strand [32], and the enzyme begins loading the RecA onto ssDNA with Chi near its end [33]. Wild-type RecBCD enzyme loads RecA only after Chi, while RecBC (lacking RecD subunit) loads RecA constitutively onto dsDNA end [34]; the *recD* mutants have no Chi activity and lack nuclease activity [35].

The RecF recombination pathway involves several proteins to process primarily replication gaps: RecQ, RecJ, RecF, RecO and RecR [36,37]. It can also repair dsDNA ends if RecBCD is inactivated and in the presence of suppressor mutations *sbcB sbcCD*. DNA at ends or gaps is unwound by RecQ protein and the 5′ end is digested by RecJ nuclease leaving 3′ ssDNA tail coated with SSB. RecF, RecO and RecR act as mediators to replace SSB with RecA on ssDNA, working independently or in pairs, but not as a stoichiometric complex [38].

Mediator proteins for synapsis also initiate HR in archaea and eukaryotes, by loading of recombinases onto ssDNA bound by SSB/RPA proteins. These include Rad52 for loading Rad51 in yeasts, BRCA2 in higher eukaryotes, a carrier protein for Rad51 monomers that displaces RPA to load Rad51 onto ssDNA. A major group of HR mediator proteins in eukaryotes and archaea, called Rad51 paralogues [26,39], promote RadA and Rad51 NPF formation and enhance synapsis. Actions of Rad51 paralogues to trigger Rad51/RadA catalysed HR is therefore another potential target for HR regulation. Recent studies in *S. cerevisiae* and *C. elegans* have provided better understanding of how Rad51 paralogues may stimulate synapsis by remodeling pre-synaptic filaments [40]. Overall, Rad51 paralogues in yeast and humans have approximately 20% sequence homology to Rad51, primarily between RecA-folds containing Walker A and B motifs. Rad51 paralogues are found in archaea (RadB), yeast (Rad55-Rad57, Csm2 and Psy3) and humans (Rad51B, Rad51C, Rad51D, XRCC2 and XRCC3). These function as multi-protein complexes that interact with Rad51 and other proteins to co-ordinate and control NPF formation and synapsis. In humans and birds, the paralogues interact into two major paralogue complexes: Rad51B-Rad51C-Rad51D-XRCC2 (BCDX2) and Rad51C-XRCC3 (CX3). These two complexes have different roles during recombination, reviewed in [26]. Rad51B-Rad51C has also been shown to stabilize Rad51 filaments [41].

*S. cerevisiae* Rad55-Rad57 heterodimers aid Rad51 in NPF formation and gain further assistance from Rad52 and the Shu complex, which comprises four proteins; Shu1, Shu2, Csm2 and Psy3. Recent bioinformatics and genetic studies have identified Shu orthologues in *C. elegans* (SWS-1) and potential candidates in humans (hSWS-1-SWSAP1). Rad55-Rad57 in yeasts, and Rfs-1 and Rip-1 in *C. elegans*, both stabilize Rad51 NPFs through physical incorporation of the paralogue complex with the recombinase in the NPF. This can enhance the effectiveness of synapsis by “opening up” the NPF for homology searches. Therefore, these recombination mediators and Rad51 paralogues act to nucleate and stabilize the NPF, promoting HR from pre-synapsis onwards. However, as mentioned earlier, unchecked or extensive HR can be dangerous as a DNA repair process, therefore synapsis is monitored and controlled to influence exactly how HR proceeds.

## 4. Disrupting Pre-synaptic Nucleoprotein Filaments: PcrA, UvrD and Fbh1

The pre-synaptic filaments assembled from coordinated actions of recombinase and mediators represent the first step toward HR. Antagonism toward NPF formation or stability would therefore discourage or avoid HR altogether. Several DNA translocases dismantle recombinase filaments, by active and passive processes: physical displacement of recombinase monomers from ssDNA, or activation of intrinsic ATPase activity within the recombinase filament causing release of monomers. The latter requires specific physical interaction between translocase and recombinase. Recently, biophysical studies have described kinetics of filament disassembly and the molecular mechanisms by which DNA translocases achieve this.

PcrA and UvrD are bacterial superfamily 1 (SF1) DNA translocases that give DNA repair phenotypes associated with roles limiting homologous recombination. Both show 3’ to 5’ translocation polarity and both disassemble RecA filaments (Figure 1A). PcrA reels in DNA in a repetitive manner when anchored at a ds-ssDNA junction similar to those found at sites of stalled replication [42]. ATP-dependent translocase activity of PcrA alone is not sufficient for removal of RecA from the filament; RecA must also be able to hydrolyse ATP and must be in an inactive ADP-bound state for filament disassembly by PcrA [43]. UvrD also removes RecA from NPFs using an ATP-dependent mechanism, observed in vitro and by live cell imaging [44,45]. Genetic analyses of UvrD have suggested a “fork-clearing role” [46] and there may be instances in which it acts as an accessory helicase to replicative helicase DnaB.

Yeast cells lacking Srs2, a yeast homologue of UvrD, display hyper-recombination phenotypes, and multiple activities of Srs2 have been shown to be effective at disassembly of Rad51 NPFs. Srs2 interacts physically with SUMO-lated PCNA, targeting it to sites of stalled replication, and also with Rad51. Physical displacement of Rad51 by 3′ to 5′ translocation of Srs2 is effective at removing Rad51 NPFs [47,48], and “scrunching” of ssDNA during this process may prevent rebinding of Rad51 monomers [49]. In addition, interaction of Srs2 with Rad51 stimulates Rad51 ATPase activity destabilizing the NPF, and therefore enhancing filament disassembly [50,51]. However, stabilization of Rad51 NPFs by Rad55-Rad57 may be effective at counteracting against the destabilizing effects of Srs2. These activities of Srs2 all antagonize extensive HR, and are thought to channel HR intermediates toward the non-crossover outcomes of SDSA [52].

A UvrD homolog in *S. pombe* and humans, F-box helicase 1 (Fbh1), also negatively regulates HR, and is unusual amongst helicases in having an F-box region that is active as a ubiquitin-ligase. Yeast (*S. pombe*) Fbh1 suppresses crossover recombination products [53] and has been implicated in controlling HR at the point of pre-synaptic NPF formation by preventing Rad51 loading onto ssDNA by Rad22, a Rad52 orthologue [54,55]. Deletion of Fbh1 from a human stem cell line resulted in a hyper-recombination phenotype [56]. Fbh1 exerts its anti-recombination effect at pre-synapsis by direct physical interaction with Rad51 and dissociation of Rad51 NPFs [53,56]. (Figure 1A). Dissociation of Rad51 requires 3′ to 5′ ssDNA translocase activity of Fbh1 [57], and human Fbh1 helicase can remodel stalled fork substrates to remove ssDNA gaps by a “fork regression” reaction [58]. This would remove ssDNA gaps by base pairing them, as the helicase migrates the fork branch point analogously to reactions described for the bacterial helicase RecG [59]. Fbh1, in the SCF complex, ubiquitylates Rad51 to negatively control synapsis, confirmed by observation of hyper-recombination phenotypes in cells expressing Rad51 that cannot be ubiquitylated [60]. Therefore the UvrD family of helicase homologs in bacteria, yeasts and higher eukaryotes provide multiple methods to control the initiation of HR at the point of pre-synaptic filaments.

## 5. Disrupting Synapsis and D-loops: RecQ and RTEL1 Helicases 

RecQ helicases were first identified in *E. coli* [61], and homologues are now characterized throughout bacteria and eukaryotes, although are mostly absent from Archaea. Recent comprehensive reviews detail the contributions made by RecQ proteins to survival and human health through various genome maintenance activities [62,63,64]. Bacterial RecQ, five human RecQ homologues (WRN, BLM, RecQ1, RecQ4 and RecQ5) and yeast RecQ proteins (Sgs1 and Rqh1) are able to unwind diverse DNA substrates, including those typical of stalled replication and intermediates formed during synapsis (e.g., D-loops) and later stages of HR (e.g., Holliday junctions). The effect of RecQ helicases is to prevent extensive HR, or to direct late HR into non-crossover products. Examples include BLM helicase-TOPOIIIα complex, which disperses Holliday junctions (“dissolution”) in an orientation that inhibits crossing over, and RecQ1 helicase, which can unwind branched DNA structures and has strand-annealing activity that may be important for converting HR into SDSA, thus reducing potential for genome instability [65]. RecQ5 helicase disrupts Rad51 from NPFs in an ATP dependent manner, facilitated by direct interaction between a C-terminal region of RECQ5 and Rad51 [66] (Figure 1A). In *C. elegans* a helicase that is not a RecQ family member, RTEL1, is synthetically lethal when deleted with RecQ5 [67]. Other RTEL1 phenotypes in *C. elegans* and mouse cells are consistent with a role that suppresses HR and maintains genome stability [67,68]. The RTEL1 mechanism seems to specifically target D-loops not Rad51 NPFs, dissociating them via its ATPase activity and thus disassembling synapsis.

## 6. HelQ and Hel308 Helicases: Mediator and Negative Regulator of Synapsis

HelQ and Hel308 are 3′ to 5′ ssDNA translocases that have been characterized in archaea, human and *C. elegans* [69,70,71,72,73], with homology to helicase domains in DNA polymerase theta [74]. *helq* and *hel308* gene deletion phenotypes and studies of fluorescent-tagged HelQ proteins are consistent with these helicases contributing to DNA repair at blocked or collapsed DNA replication forks in ways distinct from Fanconi anemia pathways [73,75,76]. HelQ and Hel308 proteins have interesting activities that both stimulate the onset of HR with Rad51 paralogues, and antagonize late stages of HR by disassembly of synaptic heteroduplex. HelQ and Hel308 interact physically with the single strand DNA binding protein RPA70 [73,75,77], which is conserved in archaea, and human HelQ interacts physically with the BCDX2 Rad51 paralogue complex [73,75]. BCDX2 seems to stabilize and promote Rad51 mediated pre-synapsis and early stages of HR [78]. A role for HelQ in this process, through its interaction with the paralogue complex, is not clear although it may help to promote synapsis of the Rad51 NPF by removing pre-bound RPA from ssDNA and allowing access to the paralogue HR mediators. Depletion of HelQ in human cell lines corresponded with a reduction in HR frequency [75]. *C. elegans* HelQ also interacts physically with Rad51 and disassembles Rad51 specifically from duplex filaments but not from ssDNA NPFs, in reactions that do not require the ATP dependent helicase activity [70]. These interesting activities of HelQ suggest that it may be a key switch that facilitates recombination dependent DNA replication, but ensures that HR does not proceed beyond the stage at which replication has been restored from a D-loop.

## 7. Homologous Recombination and the Efficiency of CRISPR Genome Editing

Homologous recombination also underpins multiple genome-editing technologies that are utilized to manipulate chromosomes and other genetic elements within cells by insertion or removal of specific DNA sequences, reviewed recently in [79]. Editing strategies are prevalent in bacteria, archaea and eukaryotes, using engineered nucleases, recombineering or application of CRISPR-Cas biology. Genome editing by “CRISPR-Cas9” has come to the fore because it offers simple precision for creating a double strand DNA break in a specific DNA sequence by RNA-DNA base pairing from a Cas9-RNA complex [80]. The Cas9 mediated editing process forms R-loops (RNA-loops), which are associated with genome instability in cells and can be used to initiate replication and recombination [81,82,83], therefore in some respects showing similarity to recombinase generated D-loops. Cas9-induced double strand DNA breaks repaired by end joining mechanisms are deleterious and therefore achieve “knockout” effects of CRISPR genome editing to study loss of function mutations. However, HR crucially underpins CRISPR genome editing for “knock-in” studies, in which the Cas9-RNA complex is accompanied by a DNA sequence for recombination into the site of the double strand break [84]. The DNA sequence for recombination is provided as duplex DNA (e.g., plasmid) or ssDNA (e.g., recombinant adeno-associated virus, AAV), and the process often has efficiencies of <1%. Inactivation of end joining systems has improved the efficiency of a CRISPR knock-in, see for example [85]. The mechanism of HR during genome editing and targeting in human, and other cell types, is still being worked out but seems to require extensive HR that avoids single-strand annealing and instead utilizes end capture and double-Holliday junction formation [86,87]. These processes may be susceptible to counter-measures and negative regulation by multiple helicases, in ways similar to when HR-dependent DNA repair. It would be useful to gain a better understanding of both precise HR mechanisms required for CRISPR editing and whether helicase anti-recombinase activities negatively impact on genome editing.
